# Implementing narrow banding imaging with dual focus magnification for histological prediction of small rectosigmoid polyps in Vietnamese setting

**DOI:** 10.1002/jgh3.13058

**Published:** 2024-05-10

**Authors:** Tien Manh Huynh, Quang Dinh Le, Nhan Quang Le, Huy Minh Le, Duc Trong Quach

**Affiliations:** ^1^ Department of Internal Medicine University of Medicine and Pharmacy at Ho Chi Minh City Ho Chi Minh City Vietnam; ^2^ GI Endoscopy Department University Medical Center Ho Chi Minh City Ho Chi Minh City Vietnam; ^3^ Department of Endoscopy Nhan Dan Gia Dinh Hospital Ho Chi Minh City Vietnam; ^4^ Department of Histology‐Embryology and Pathology University of Medicine and Pharmacy at Ho Chi Minh City Ho Chi Minh Vietnam

**Keywords:** colorectal polyp, dual focus, magnification, narrow‐band imaging, Vietnam

## Abstract

**Background and Aim:**

Small rectosigmoid colorectal polyps (<10 mm) are prevalent, with a low prevalence of advanced neoplastic lesions. The “diagnose‐and‐leave” strategy, employing narrow band imaging (NBI), is gaining popularity for its safety and cost‐effectiveness by reducing polypectomy complications and minimizing histopathology expenses. This study assessed the diagnostic efficacy of NBI with dual focus (DF) magnification for real‐time neoplastic prediction of rectosigmoid polyps and explored the feasibility of implementing this strategy in Vietnam.

**Methods:**

In a prospective single‐center study, 307 rectosigmoid polyps from 245 patients were analyzed using three consecutive endoscopic modes: white light endoscopy (WLE), NBI, and NBI‐DF. Endoscopists assessed polyps for size, location, macroscopic shape, optical diagnosis, and confidence levels before histopathological evaluation. High confidence was assigned when the polyp exhibited all features of a single histology type. Predictions were compared with final histopathology results.

**Results:**

Of the total, 237 (77.2%) were diminutive (≤5 mm) polyps, and 18 (5.8%) were advanced neoplastic lesions. WLE + NBI and WLE + NBI + NBI‐DF exhibited significantly higher accuracy compared to WLE (88.6% and 90.2% *vs* 74.2%, *P* < 0.01). For diminutive polyps, the DF mode significantly increased the rate of high‐confidence optical diagnoses (89.1% *vs* 94.9%, *P* < 0.001). WLE + NBI + NBI‐DF demonstrated high sensitivity (90.1%), specificity (95.5%), and negative predictive value (93.4%) in high‐confidence predictions, enabling the implementation of the “diagnose‐and‐leave” strategy. This approach would have reduced 58.2% of unnecessary polypectomies without missing any advanced neoplastic lesions.

**Conclusion:**

NBI and DF modes provide accurate neoplastic predictions for rectosigmoid polyps. For diminutive polyps, DF magnification improves the confidence level of the optical diagnosis, allowing the safe implementation of the “diagnose‐and‐leave” strategy.

## Graphical abstract

This study assessed the diagnostic efficacy of NBI with dual focus (DF) magnification for real‐time neoplastic prediction of diminutive rectosigmoid polyps. WLE + NBI and WLE + NBI + NBI‐DF exhibited significantly higher accuracy compared to WLE. The DF mode significantly increased the rate of high‐confidence optical diagnoses. WLE + NBI + NBI‐DF demonstrated high sensitivity (90.1%), specificity (95.5%), and negative predictive value (93.4%) in high‐confidence predictions, enabling the implementation of the “diagnose‐and‐leave” strategy.

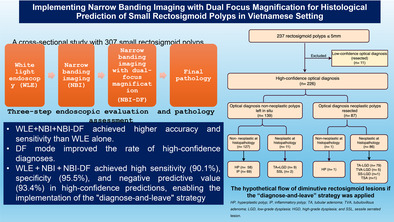

## Introduction

The application of white light endoscopy (WLE) in isolation is inadequate for discriminating between neoplastic and nonneoplastic polyps, which may lead to unnecessary excision of many lesions. Enhanced imaging endoscopy has been recognized as advantageous for assessing colorectal polyps, with real‐time optical diagnosis gaining increasing acceptance and endorsement by guidelines.^1^ Given the low likelihood of high‐risk lesions in the rectosigmoid segment, a recent novel approach, “diagnose‐and‐leave,” is being demonstrated as a potentially cost‐effective strategy for managing rectosigmoid polyps. This minimizes histopathological costs and polypectomy complications.^1^, ^2^, ^3^ Furthermore, the application of this approach should be contingent on an optical diagnosis meeting the accuracy thresholds prescribed by the Preservation and Incorporation of Valuable Endoscopic Innovation document, as outlined by the American Society of Gastrointestinal Endoscopy (ASGE) and the European Society of Gastrointestinal Endoscopy (ESGE).^1^, ^4^ The “diagnose‐and‐leave” strategy for diminutive (≤ 5 mm) colorectal lesions is clinically acceptable if real‐time colonoscopy can achieve at least 90% sensitivity and 80% specificity (ESGE) and a negative predictive value (NPV) of at least 90% (ASGE) for high‐confidence endoscopic prediction of diminutive colorectal neoplasia in the rectosigmoid region, with histopathology serving as the gold standard.^1^, ^5^ Recently, an innovative endoscopic system with novel features, such as narrow band imaging (NBI) and dual focus (DF) magnification mode, has been introduced. This system allows users to adjust magnification for close‐up examination easily. With this optical advancement, users can choose between two focus settings, achieving a 45‐fold magnification on a 26‐inch monitor. Furthermore, by utilizing a 1.4‐ to 2.0‐fold electronic zoom function, a maximum magnification of 90‐fold can be attained.^6^, ^7^ Several studies have shown that NBI and the DF mode are highly effective at predicting colorectal neoplastic lesions.^3^, ^7^, ^8^, ^9^, ^10^, ^11^, ^12^ While these advanced diagnostic techniques are gaining popularity in Vietnam, more systematic data on their  perfomance in predicting colorectal polyp histology are needed. Our previous study demonstrated the high diagnostic yield of NBI‐DF for small colorectal polyps.^13^ Therefore, we performed a further study to assess the diagnostic yield of NBI and NBI‐DF for predicting small rectosigmoid neoplastic polyps and the feasibility of employing the “diagnose‐and‐leave” strategy.

## Methods

### Study design, setting, and patient recruitment

This cross‐sectional, single‐center observational study was conducted at the City General Hospital in Ho Chi Minh City, Vietnam, from September 2020 to May 2021. We gathered data, building upon the findings of a previous study, with a more focused emphasis on the rectosigmoid colon.^13^ The study's inclusion criteria consisted of the following: (1) individuals aged 18 years and above who underwent elective colonoscopy (screening, surveillance, or diagnostic workup) in consecutive order, having provided informed consent before participation; (2) detection of small colorectal polyps (<10 mm in size) in the rectosigmoid colon; and (3) retrieval of polyps for histological examination. Exclusion criteria included: (1) individuals with polyps larger than 10 mm or located outside the rectosigmoid colon; (2) those with suboptimal bowel preparation, indicated by a Boston Bowel Preparation Scale score less than 6 and incomplete cecal intubation; (3) individuals with contraindications to total colonoscopies, such as severe heart failure, severe respiratory failure, shock, or similar conditions; and (4) individuals diagnosed with synchronous colorectal cancer, inflammatory bowel disease, polyposis syndrome, or pregnancy.

### Data collection

Patient demographic data, including age and gender, were collected.

To prepare for the colonoscopic procedures, each patient ingested 1.5–2 L of polyethylene glycol solution and 1000 mg of simethicone. Colonoscopies were performed by two experienced endoscopists (QDL and NQL) who had previously conducted more than 1000 procedures using the NBI and DF modes. The evaluation utilized the Olympus EVIS EXERA III series 190, which features NBI endoscopy in DF mode, a light source, image processing, and a flexible colonoscope. A push‐button system enabled seamless switching between NBI and WLE. The NBI‐DF mode employed a 1.4× electronic zoom and a transparent cap for precise focus.

**Figure 1:**
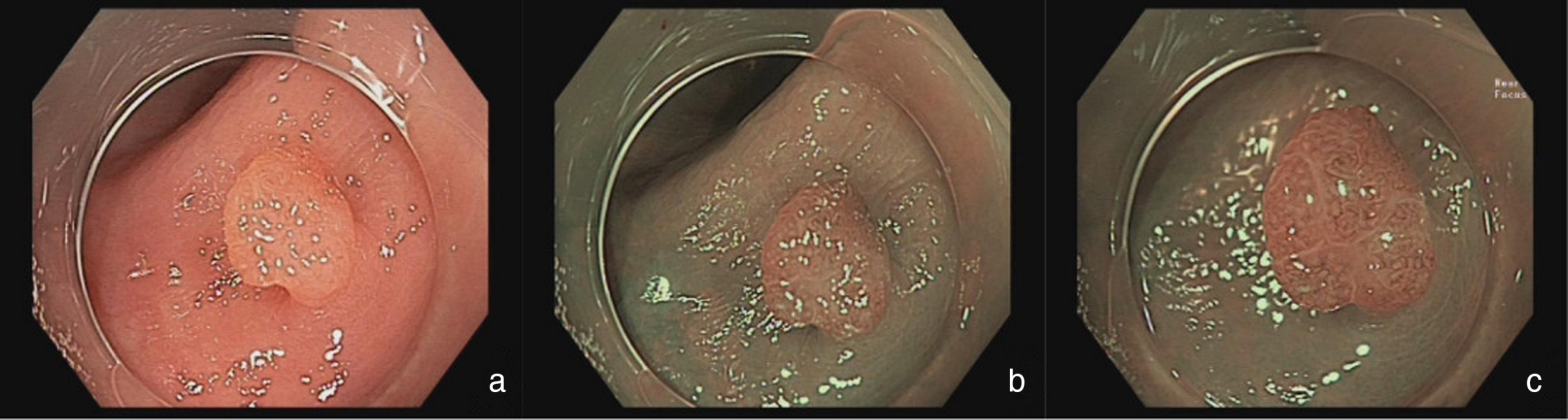
A sigmoid colon lesion (4 mm, 0‐Is) in WLE (a) was classified as NICE type 1 in the WLE+ NBI and WLE + NBI + NBI‐DF modes and was characterized by pale color, absence of vessels, and a black dot pattern (b, c). Histopathology: hyperplastic polyp.

**Figure 2:**
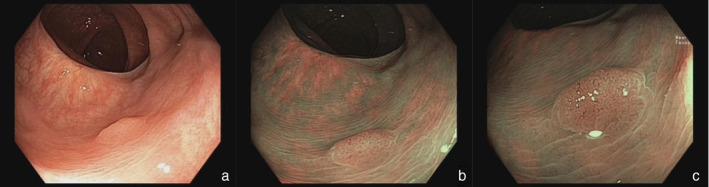
A rectal lesion (5 mm, 0‐Is) was detected via WLE (a). It is classified as NICE type 2 in NBI and NBI‐DF modes. It is characterized by a brown color and thick brown vessels surrounding tubular, oval, or pentagonal pits (b, c). Histopathology revealed a low‐grade tubular adenoma.

Colonoscopies were performed using WLE for the entire colon, with the endoscope inserted into the Bauhin valve and the ileocecal angle. During endoscopic tube withdrawal, the endoscopists closely observed the small rectosigmoid colorectal polyps. In the WLE mode, QDL and NQL assess the polyp according to the number, location, size (compared to the open jaws of the biopsy forceps or snare), gross morphology according to the Paris classification, and histological prediction.^14^ Lesion size was assessed by comparing the polyp dimensions to those of the biopsy forceps (2.3 mm closed, ENDO‐FLEX) and polypectomy snare (10 mm open, SnareMaster®, Olympus). Based on previous reports, polyps were classified as either nonneoplastic or neoplastic based on their surface patterns: polyps were classified as either nonneoplastic (circular white pits, very fine capillary network bordering the pit) or neoplastic (circular/oval/linear/cerebri form pits, dendrite or gyrus‐like pits, or an irregular arrangement or loss of pits).^15^ Subsequently, the polyp was examined in NBI mode and NBI‐DF mode using the NICE classification to classify the rectal polyp based on its color, blood vessels, and surface characteristics (Table S1, Supporting information).^10^ Afterward, the endoscopist assessed the confidence of the prediction results as either low or high. If the polyp exhibits all the features of a single histology type, the prediction was deemed to have “high confidence.” Any uncertainty or doubt about any feature results in a prediction with low confidence.^5^ An independent observer (TMH) recorded the diagnosis at each stage. A simethicone‐based washing system was used during the endoscopy to enhance foam dissolution and surface cleaning, improving the assessment process. All polyps were removed en bloc through snare polypectomy or biopsy and sent for histological evaluation. In cases of multiple rectal lesions with NICE type 1 suspected to be hyperplastic, endoscopists were not obligated to remove all of them. Instead, they selectively removed or sampled only the first five, prioritizing the largest lesions. The procedure was illustrated through Video S1 and Figures 1 and 2.

### Histopathological investigations

After resection, each lesion was collected in individually numbered containers. The specimens were stained with standard hematoxylin and eosin (H&E) and analyzed by an expert gastrointestinal pathologist (HML) blinded to all the endoscopic images and assessments. Lesions were categorized based on the 2019 World Health Organization (WHO) classification.^15^ Neoplastic lesions were classified as adenomas, sessile serrated lesions (SSLs), or adenomatous polyps, while nonneoplastic lesions were classified as inflammatory polyps, hyperplastic polyps, or normal colorectal mucosa. High‐risk neoplastic lesions included high‐risk adenomas with villous components and SSLs with dysplasia.

### Statistical analysis

This study aimed to assess the accuracy of optical diagnosis for predicting neoplastic lesions in the rectum and sigmoid via comparison with histopathology, which is considered the reference standard. The visual diagnosis was evaluated by creating 2 × 2 contingency tables to calculate sensitivity, specificity, predictive values, and their corresponding 95% confidence intervals (CIs). Categorical variables are expressed as proportions or percentages, and continuous variables are presented as means (± standard deviation, SD). Paired proportions were compared using McNemar's test, with statistical significance set at *P* < 0.05 (two‐sided). SPSS statistical software (version 23.0, IBM Corp, Armonk, NY, USA 2012) was used to analyze the data.

### Ethical considerations

This study's protocol received approval from the Institutional Review Board of the University of Medicine and Pharmacy at Ho Chi Minh City, Vietnam. Before the examination, all patients provided written informed consent.

## Results

### Patient clinical and pathological profile characteristics

From August 2020 to May 2021, 324 polyps were collected from 245 consecutive patients who met the sample selection criteria. Histopathological examination was performed on 307 polyps after excluding 17 incompletely sampled polyps. The characteristics of patients and lesions, including clinical, endoscopic, and pathological aspects, are outlined in Table 1. The study focused only on rectosigmoid polyps measuring less than 10 mm, so the polyp and adenoma detection rates were not calculated. Most polyps (77.2%) were diminutive and exhibited 0‐IIa morphology (59%). The most common histopathological type was tubular adenoma (45.9%), which was primarily low‐grade dysplasia (48.2%), and 97.7% of cases did not have dysplasia. SSLs accounted for 1.3% of the lesions (4/307 polyps). Neoplastic lesions measuring less than ≤5 mm and 6–9 mm in size accounted for 49.1% (213/307 polyps) and 78.1% (82/307 polyps) of the patients, respectively. Notably, no cases of cancer were detected.

**Table 1:** Clinical characteristics of the patients and polyps

Characteristics	*n*	%
Characteristic of patient		
Age (X ± SD, min, max)	59.8 ± 13.2	
Male/female	141/104	
Characteristic of polyp		
*N*	307 polyp	100
Size, *n* (mm)	3.8 ± 2.3	
Size group		
≤5 mm	237	77.2
6–9 mm	70	22.8
Location		
Rectum	108	35.2
Sigmoid colon	199	64.8
Method of endoscopic removal		
Biopsy forceps	217	70.7
Cold snare polypectomy	32	10.4
Hot snare polypectomy	58	18.9
Morphology		
Sessile (0‐Is)	117	38.1
Flat elevated (0‐IIa)	181	59.0
Pedunculated (0‐Ip)	9	2.9
Histological findings		
Inflammatory	76	24.8
Hyperplastic	75	24.4
Sessile serrated lesion	4	1.3
Tubular adenoma	139	45.9
Tubulovillous adenoma	12	3.9
Villous adenoma	0	0
Revised Vienna classification		
Negative for neoplasia	152	49.5
Indefinite for neoplasia	0	0
Mucosal low‐grade neoplasia	148	48.2
Mucosal high‐grade neoplasia	7	2.3
Submucosal invasion by carcinoma	0	0

### Relationship between real‐time optical diagnosis and histological results

The predictive rates of neoplastic lesions were 97 out of the 157 patients (61.1%) who underwent WLE, 138 out of the 157 patients (86.8%) who underwent WLE + NBI, and 144 out of the 157 patients (90.6%) who underwent WLE + NBI + NBI‐DF (Table 2). Within the subset of neoplastic polyps, 12.1% were erroneously categorized as NICE type 1 when using WLE + NBI and 8.2% when applying WLE + NBI + NBI‐DF. Similarly, among the nonneoplastic polyps, 10.6% were misidentified as NICE 2 under WLE + NBI, and 11.3% were misidentified under WLE + NBI + NBI‐DF. The greatest agreement was observed between histopathology and the prediction based on WLE + NBI + NBI‐DF (91.2%, kappa = 0.823), followed by WLE and NBI (89.6%, kappa = 0.791). The lowest agreement was found with WLE alone (63.2%, kappa = 0.272). The clinicopathological features of 18 high‐risk lesions are shown in Table S2. Notably, all high‐risk lesions were accurately classified as NICE 2 and made with high confidence.

**Table 2:** Association between endoscopic neoplastic prediction and histological findings

Modality	Endoscopic prediction	Histopathology result		Agreement	Expected agreement	Kappa
		Neoplastic(*n* = 157)	Nonneoplastic (*n* = 151)			
WLE	Neoplastic	97	134	63.2%	49.4%	0.272
	Nonneoplastic	60	16			
WLE + NBI	NICE 1	16	135	89.6%	50.1%	0.791
	NICE 2	140	16			
WLE + NBI + NBI‐DF	NICE 1	11	135	91.2%	50.0%	0.823
	NICE 2	145	16			

NBI, narrow banding imaging; NBI‐DF, narrow banding with dual focus magnification; WLE, white light endoscopy.

### Diagnostic performances for histology predictions after adding NBI and NBI‐DF

Using the WLE + NBI and WLE + NBI + NBI‐DF modes significantly improved the efficacy of predicting neoplastic lesions (Table 3). When NBI was combined with WLE, there was a substantial increase in sensitivity compared to that of WLE alone (89.8% *vs* 61.8%, *P* < 0.001) and a greater NPV (89.4% *vs* 68.0%, *P* < 0.001). Furthermore, the addition of NBI‐DF significantly enhanced diagnostic accuracy, particularly sensitivity (93.6% *vs* 89.8%), NPV (92.5% *vs* 89.4%), and overall accuracy (90.2% *vs* 88.6%). However, there was no significant difference in diagnostic performance between WLE + NBI and WLE + NBI + NBI‐DF (*P* = 0.13).

**Table 3:** Diagnostic yield of WLE, NBI, and NBI‐DF for predicting small neoplasms

Diagnostic performance	WLE	WLE + NBI	WLE+ NBI + NBI‐DF	p1	p2
Sensitivity	61.8	89.8	93.6	<0.001	0.13
(95% CI)	53–68.6	83.8–94.0	87.7–96.4		
Specificity	89.2	89.4	89.4	1	1
(95% CI)	83–93.7	83.4–93.8	83.4–93.8		
PPV	85.4	84.6	90.1	1	1
(95% CI)	79–90.7	84.6–93.3	85.1–93.5		
NPV	68.0	89.4	92.5	<0.001	0.13
(95% CI)	63.5–72.3	84.1–93.1	87.4–95.6		
Accuracy	74.6	88.6	90.2	<0.001	0.13
(95% CI)	69.3–79.4	85.6–92.8	87.5–94.1		

CI, confidential interval; NBI, narrow banding imaging; NBI‐DF, narrow banding with dual focus magnification; p1, McNemar test (WLE *vs* NBI); p2, McNemar test (NBI *vs* NBI‐DF); WLE, white light endoscopy.

### Diagnostic yield of WLE + NBI and WLE + NBI + NBI‐DF for characterizing histological lesions in the rectosigmoid colon according to polyp size with high confidence

Tables 4 and 5 present an overview of the optical diagnostic performance and the rate of high confidence stratified by polyp size.

**Table 4:** High‐confidence rates in WLE + NBI and WLE + NBI + NBI‐DF stratified by polyp size

	Size group	WLE + NBI	WLE + NBI + NBI‐DF	*P*
HC rate (%)	6–9 mm	66 (94.3)	68 (97.1)	0.5
	≤5 mm	211 (89.1)	226 (94.9)	<0.001

HC, high confidence; NBI, narrow banding imaging; NBI‐DF, narrow banding with dual focus magnification; p, McNemar test; WLE, white light endoscopy.

**Table 5:** Diagnostic yield of optical diagnosis, made with high confidence

Size group	Diagnostic performance	WLE + NBI	WLE + NBI + NBI‐DF	*P*
6–9 mm	Sensitivity	100	100	1
	(95% CI)	93.5–100	93.7–100	
	Specificity	36.4	36.4	1
	(95% CI)	10.9–69.2	10.9–69.2	
	PPV	88.7	89.1	1
	(95% CI)	83.4–92.5	83.9–92.7	
	NPV	89.4	100	1
	(95% CI)	76.4–95.6		
	Accuracy	89.4	89.7	1
	(95% CI)	79.4–95.6	79.9–95.8	
≤ 5 mm	Sensitivity	87.3	90.1	0.06
	(95% CI)	71.5–88.3	82.1–95.4	
	Specificity	95.4	95.5	1
	(95% CI)	90.4–98.3	90.5–98.3	
	PPV	92.0	93.2	1
	(95% CI)	83.9–96.2	86–96.7	
	NPV	92.7	93.4	0.06
	(95% CI)	87.7–95.8	86.2–94.7	
	Accuracy	92.5	93.3	0.06
	(95% CI)	88.0–95.6	87.7–95.2	

CI, confidence interval; HC, high confidence; NBI, narrow banding imaging; NBI‐DF, narrow banding with dual focus magnification; p1, McNemar test (NBI *vs* NBI‐DF); WLE, white light endoscopy.

Regarding the confidence level, in the 6–9 mm group, adding the DF mode did not significantly improve the confidence rate (94.3 *vs* 97.1, *P* = 0.5). In addition, for diminutive polyps, the DF mode significantly improved the proportion of high‐confidence optical diagnoses (89.1% *vs* 94.9%, *P* < 0.001).

For polyps ≤5 mm, with high confidence predictions, WLE + NBI + NBI‐DF had a slightly greater sensitivity (90.1% *vs* 87.3%), specificity (95.5% *vs* 95.4%), and NPV (92.7% *vs* 93.4%) than did WLE + NBI. However, it is essential to note that the observed differences were not statistically significant.

**Figure 3:**
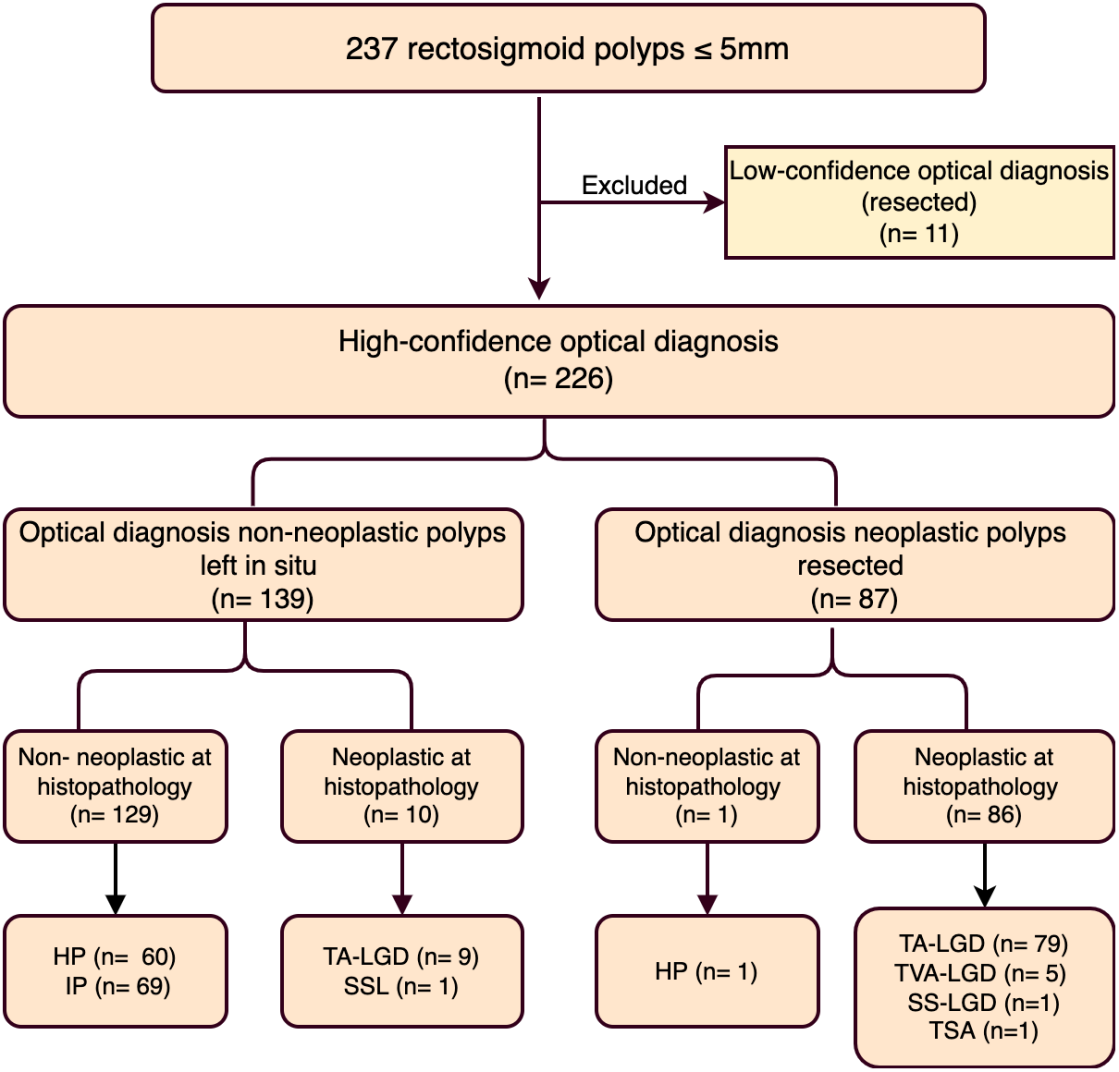
The hypothetical flow of diminutive rectosigmoid lesions if the “diagnose‐and‐leave” strategy was applied. HGD, high‐grade dysplasia; HP, hyperplastic polyp; IP, inflammatory polyp; LGD, low‐grade dysplasia; SSL, sessile serrated lesion; TA, tubular adenoma; TVA, tubulovillous adenoma.

WLE + NBI and WLE + NBI + NBI‐DF met the ASGE recommendation, with an NPV > 90% for predicting neoplastic polyps. However, for the ESGE recommendation, only WLE + NBI + NBI‐DF achieved a sensitivity >90% and specificity >80% in predicting neoplastic polyps. We devised the subsequent flowchart to document the implementation of the “diagnose and leave” strategy using NBI‐DF, as it meets the ASGE and ESGE guidelines (Fig. 3). When implemented, this approach led to the removal of 99 out of 237 (41.8%) polyps with low confidence (11 polyps) or those predicted as neoplastic lesions (87 lesions). Additionally, in 10 out of 98 (10.2%) polyps, neoplastic lesions were misdiagnosed, including nine low‐grade tubular adenomas and one SSL. However, with the implementation of this strategy, no high‐risk lesions could be missed.

**Figure 4:**
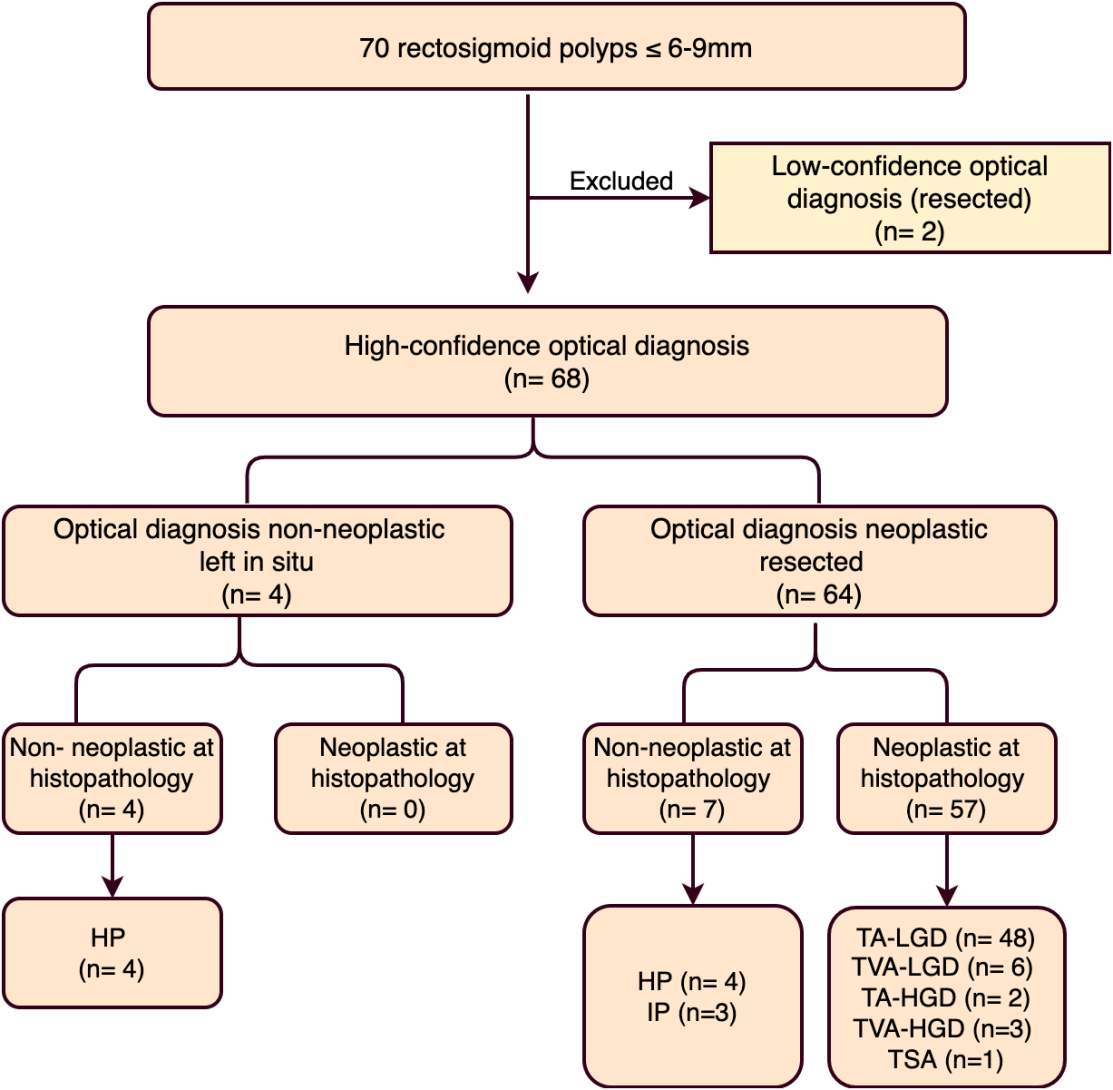
The hypothetical flowchart of 6–9 mm rectosigmoid lesions if the “diagnose‐and‐leave” strategy was applied. HGD, high‐grade dysplasia; HP, hyperplastic polyp; IP, inflammatory polyp; LGD, low‐grade dysplasia; SSL, sessile serrated lesion; TA, tubular adenoma; TVA, tubulovillous adenoma.

For 6–9 mm polyps with high confidence predictions, WLE + NBI and WLE + NBI + NBI‐DF demonstrated 100% sensitivity, 36.4% specificity, and an NPV ranging from 89.4% to 100% for predicting neoplastic histology. Implementing “diagnose‐and‐leave” strategy for this size group would have only avoided 5.7% (4/70 lesions) of the polypectomies (Fig. 4).

## Discussion

To the best of our knowledge, our study is the first to investigate the utility of NBI and DF magnification for real‐time neoplastic histology prediction of small rectosigmoid polyps in the Vietnamese population. We also evaluated the feasibility of adopting the “diagnose‐and‐leave” strategy following recommendations from the ASGE and ESGE. First, we reaffirmed the effectiveness of WLE + NBI and WLE + NBI + NBI‐DF in predicting neoplastic histopathology of rectosigmoid polyps compared with WLE alone. Second, WLE + NBI + NBI‐DF achieved a high NPV, sensitivity, and specificity that met the ESGE and ASGE thresholds for this strategy, potentially reducing unnecessary polypectomies for diminutive rectosigmoid lesions by approximately 60%.

Regarding the diagnostic performance of WLE + NBI and WLE + NBI + NBI‐DF, during real‐time endoscopy, WLE + NBI + NBI‐DF yielded the highest kappa value when compared to histopathological standards (0.823), surpassing WLE + NBI (0.791), and WLE (0.272). Many studies have demonstrated the high diagnostic yield of NBI and NBI‐DF for optical diagnosis of the rectosigmoid colon.^8^, ^11^, ^16^ The difference in our study is that we employed consecutive three‐step evaluations in real time with our local team in the Vietnamese setting. The remarkable outcomes in this study were attributable to a combination of critical factors, including (1) novel enhanced endoscopy with easy‐to‐use magnification mode, (2) a well‐skilled endoscopist who had more than 10 years of experience, (2) excellent bowel preparation with intensive polyp surface cleaning before prediction, and (3) universal and well‐compatible, easy‐to‐use classification (NICE classification).^13^ Furthermore, the addition of the DF mode improved the performance compared to NBI alone in sensitivity (89.8 *vs* 93.6, *P* = 0.13) and accuracy (88.6 *vs* 90.2, *P* = 0.13), but this difference was not statistically significant (*P* = 0.13). Thus, regarding small neoplastic prediction, WLE + NBI may be considered noninferior to NBI‐DF in the rectosigmoid segment. This nonsignificant improvement could be due to the endoscopist's expertise or limitations in magnification (approximately ×65) compared to other colonoscopies (×100 to ×100–125). Additionally, the 190 series may offer optical information or additional details that may lead to interpretation confusion.^17^

**Figure 5:**
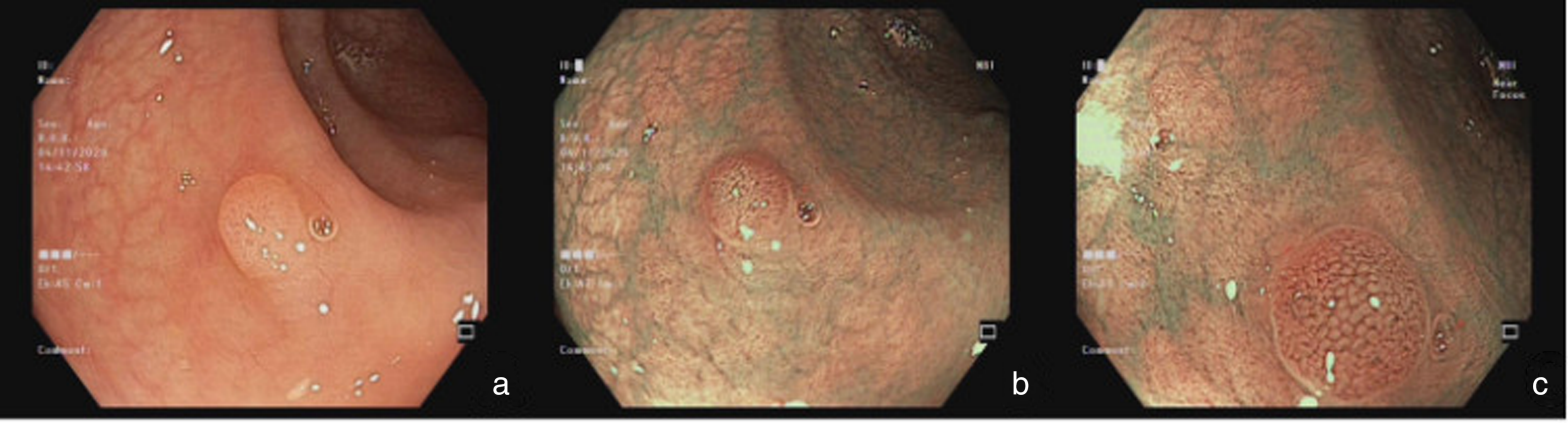
A lesion (5 mm, 0‐Is) in the sigmoid colon was initially identified as neoplastic in WLE and NICE type 2 patients with low confidence in NBI mode. However, with the application of dual focus mode, the lesion was confidently classified as NICE type 2 (C). Histopathology revealed a low‐grade tubular adenoma.

However, regarding the level of confidence, adding the DF mode aided endoscopists in lesion characterization and a notable increase in the percentage of high‐confidence interpretations for polyp histology, even for polyps measuring ≤5 mm, rising from 89.1% with NBI alone to 94.9% with NBI + NBI‐DF (*P* < 0.001; Table 4 and Fig. 5).

Prior studies have demonstrated that the use of DF mode or magnifying endoscopy can improve the rate of high‐confidence optical diagnosis.^8^, ^18^ The improvement in confidence level was due to magnification (65×) coupled with increased resolving power (1.56×) in the near‐focus view and the prefreeze function, which identifies the sharpest image from the earlier 4–29 frames. These factors led to fewer image capture attempts for high‐quality photos and higher subjective image quality scores.^17^

Regarding the feasibility of the “diagnose‐and‐leave” strategy, we analyzed 6–9 mm and 5 mm group sizes. In the 6–9 mm group, WLE + NBI + NBI‐DF met the ASGE's NPV threshold but did not satisfy the ESGE criteria and had a modest 5.7% reduction in polypectomies. Additionally, for diminutive polyps, WLE + NBI + NBI‐DF met the stringent criteria of both ESGE and ASGE, demonstrating a substantial overall reduction of 58.9%. Among the 237 diminutive rectosigmoid lesions evaluated with high confidence, only 11 were incorrectly identified as nonneoplastic lesions: 9 were TA‐LGDs, and one non‐dysplastic SSL.

Similarly, previous studies have demonstrated that both NBI and NBI‐DF can meet the criteria of ASGE or ESGE^7^, ^8^, ^19^ (Table 6). Additionally, the meeting of ASGE and ESGE thresholds of WLE + NBI + NBI‐DF may be due to the improvement of the rate of high‐confidence lesions in optical diagnosis.

**Table 6:** Diagnostic performance of NBI and the DF mode for diminutive rectosigmoid polyps

Author	Country	Study type	Number of rectosigmoid lesions	Endoscopic diagnostic criteria	Number of endoscopists and NBI training program	Confidence level improvement	Neoplastic prevalence ≤ 5 mm (%)	Sensitivity (%)		Specificity (%)		NPV (%)	
								NBI	NBI‐DF	NBI	NBI‐DF	NBI	NBI‐DF
Singh^23^	Australia	CS	40	Sano classification	NA	NA	60	NA	100	NA	100	NA	100
Wallace^16^	USA	RCT	224	NICE classification	7—Ex vivo images training modules	NA	20–25	85.7	75.0	91.6	96.1	96	97
Kaltenbach^8^	USA	RCT	401	NICE classification	5—a Learning Management System	Yes	30–36.5	96.7–92.3	95.5–77.8	79.2–90.2	83.6–81.1	93.6	96.6
Kuruvilla^24^	Australia	CS	80	Kudo Modified Sano classification	3—15 years of experience in colonoscopy with extensive use and knowledge of Kudo and Sano classification	NA	57.5	NA	94	NA	97	NA	96
Our study	Vietnam	CS	530	NICE classification	2—experience in >1000 NBI‐colonoscopy/year	Yes	49.1	87.3	90.1	95.4	95.5	92.7	93.4

CS, cross‐sectional study; NA, not available; NBI, narrow banding imaging; NBI‐DF, narrow banding imaging with dual focus mode; RCT, randomized control trial.

**Figure 6:**
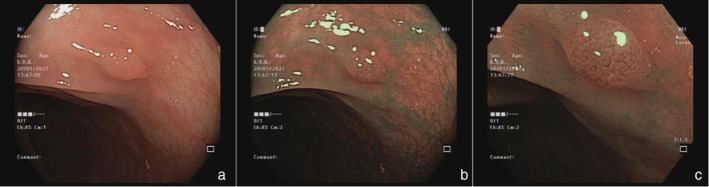
A lesion (3 mm, 0‐IIa) in the sigmoid colon. Initially, the lesions were classified as nonneoplastic according to the WLE mode (A) or NICE type 1 according to the NBI and NBI‐DF modes. A large open pit, a hallmark of serrated lesions, was evident in the NBI‐DF images. The confirmed histopathology was a sessile serrated lesion.

Furthermore, the limitation of the NICE classification was that hyperplastic polyps and SSLs could not be differentiated within the NICE 1 category. However, we did not use the Workgroup Serrated Polyps and Polyposis classification due to the low proportion of SSL (1.3%). In the rectosigmoid colon, the proportion of NICE type 1 lesions ranging from 1 to 5 mm of SSL is less than 2%, indicating that the NPV of NICE type 1 features for both adenoma and SSL is high.^20^ The sole distinguishing feature within the NICE classification between these two types is the presence of large open pits in the SSL. However, the DF mode may facilitate this differentiation (Fig. 6).

The misclassification could also be related to endoscopist‐related factors, such as poor photo documentation, limitations in classification systems, and incomplete histology. In real‐time, photo documentation can be rendered by a bowel movement, the experience of an endoscopist. Furthermore, some adenomas are mistakenly classified based on an initial assessment of color, similar to the background mucosa under WLE and NBI.^21^ Furthermore, 15% of lesions measuring 1–3 mm, identified as adenomas under NBI, are pathologically diagnosed as normal tissue, regardless of the removal method (snaring or forceps), revealing a discrepancy between endoscopic and pathological assessments.^22^ However, we did not assess the accuracy of our pathologists; therefore, the effect on the outcome due to variation in pathological diagnosis accuracy could not be assessed.

This study's prospective data collection and real‐time optical diagnosis have significant implications for clinical practice. The NBI and DF models demonstrate promising potential for accurately identifying and excluding diminutive neoplastic rectosigmoid lesions, supporting the adoption of the “diagnose‐and‐leave” strategy. This approach can effectively reduce procedural risks and pathological costs, diverging from the costly practice of biopsying or resecting all lesions. However, successfully implementing this strategy requires standardized training, accreditation, patient acceptance, and the availability of endoscopy systems. Moreover, introducing optical diagnosis to endoscopists accustomed to WLE presents a challenge. Concerns such as legal liability and financial disincentives may hinder its adoption. Nevertheless, inclusion in clinical guidelines and developing a reimbursement strategy could facilitate its integration into clinical practice.

Our study has several limitations. First, in our study, the lower limit value of NPV and sensitivity (95% CI) did not meet the threshold, which were partially due to our small sample size and a lower prevalence of non‐neoplastic cases as multiple hyperplastic lesions were not resected due to ethical concerns. To obtain more comprehensive insights, conducting more extensive, multicenter studies involving a broader range of less experienced and community physicians with a larger sample size is imperative. Second, the study did not assess individual patient risk or the proportion of patients with serrated adenomas or their associated risks due to the limitations of the NICE classification system, which potentially impacts the predictive outcome. Third, all procedures were conducted by a single expert, potentially limiting generalizability. We typically follow consecutive phases in our daily practice, including WLE, NBI, and NBI‐DF. Additionally, we did not opt for randomized controlled trials. Furthermore, we did not compare our findings with those of WLE with DF magnification (WLE‐DF) due to the absence of a validated classification system for predicting polyp histology using white light. Finally, the “resect and discard” strategy was excluded due to legal requirements, the absence of a Vietnamese surveillance interval policy, or the common occurrence of early colorectal cancers in the rectosigmoid.

In summary, our study underscores the excellent diagnostic performance of both NBI and NBI‐DF for small neoplastic polyps in the rectosigmoid segment. Adopting the “diagnose‐and‐leave” strategy with NBI‐DF can significantly decrease unnecessary procedures and associated costs by up to 58.2% without compromising the detection of high‐risk lesions. This approach is especially beneficial in low‐resource settings such as Vietnam, where traditional optical zoom techniques may be constrained.

## Consent

Patient consent was obtained.

## Supporting information


**Video S1.** Real‐time endoscopic optical diagnosis of rectosigmoid colorectal polyp. NBI, narrow band imaging; NBI‐DF, narrow banning imaging with dual focus mode; WLE, white light endoscopy.

**Table S1.** Narrow‐band imaging international colorectal endoscopic classification.
**Table S2.** Characteristics of high‐risk polyps (*N* = 18).

## Data Availability

The patients’ data used to support the findings of this study are available from the corresponding author upon request.
