# Supplementary material for: Implementing narrow banding imaging with dual focus magnification for histological prediction of small rectosigmoid polyps in Vietnamese setting
Source: JGH Open. 2024 May 10;8(5):e13058. doi: 10.1002/jgh3.13058 (PMC11087732; doi:10.1002/jgh3.13058)
Supplement: Supplementary file 2 — Table S1. Narrow‐band imaging international colorectal endoscopic classification. Table S2. Characteristics of high‐risk polyps (N = 18). [file JGH3-8-e13058-s002.docx]

**Supplementary table 1. Narrow-band imaging International Colorectal Endoscopic Classification**

|  | **Type 1** | **Type 2** | **Type 3** |
| --- | --- | --- | --- |
| Color | Same or lighter than background | Browner relative to background (verify color arises from vessels) | Brown to dark brown relative to background; sometimes patchy whiter areas |
| Vessels | None or isolated lacy vessels may be present coursing across the lesion | Brown vessels surrounding white structures[^2^](https://www.ncbi.nlm.nih.gov/pmc/articles/PMC4265957/table/T1/?report=objectonly#T1FN1) | Has area(s) of disrupted or missing vessels |
| Surface Pattern | Dark or white spots of uniform size, or homogeneous absence of pattern | Oval, tubular, or branched white structures surrounded by brown vessels | Amorphous or absent surface pattern |
| Most likely pathology | Hyperplastic | Adenoma | Deep submucosal invasive cancer |

**Supplementary table 2. Characteristics of high-risk polyps (N=18)**

| **Charateristic** | **N (%)** |
| --- | --- |
| **Size** |  |
| ≤ 5mm | 9 (50) |
| 6-9mm | 9 (50) |
| **Paris classification** |  |
| 0-Ip | 3 (16.7) |
| 0-Is | 14 (77.8) |
| 0-IIa | 5 (5.5) |
| **Location** |  |
| Rectum | 8 (44.4) |
| Sigmoid | 10 (55.6) |
| **WLE** |  |
| Non-neoplastic | 3 (16.7) |
| Neoplastic | 15 (83.3) |
| **WLE+NBI** |  |
| NICE 1 | 0 (0) |
| NICE 2 | 18 (100) |
| **WLE+NBI+NBI-DF** |  |
| NICE 1 | 0 (0) |
| NICE 2 | 18 (100) |
| **High- confidence level** |  |
| NBI | 18 (100) |
| NBI-DF | 18 (100) |
| **Histology** |  |
| Low grade tubulovilluous | 10 (55.6) |
| High-grade dysplasia tubulovilluous | 2 (11.1) |
| High-grade dysplasia tubular | 5 (27.8) |
| Serrated lesion with high-grade dysplasia | 1 (5.5) |
